# Modification of Domestic Animal Lameness Scales for Use in Asiatic Black Bears *(Ursus thibetanus)*

**DOI:** 10.3390/ani13213302

**Published:** 2023-10-24

**Authors:** Mandala Hunter-Ishikawa, Jamie Y. Nakatani, David S. Miller

**Affiliations:** 1 Animals Asia Foundation, Hong Kong Post Box P.O. Box 374, General Post Office, Hong Kong; 2 Animal Behavior Graduate Group, School of Veterinary Medicine, University of California, Davis, One Shields Avenue, Davis, CA 95616, USA; 3 Miller Veterinary Services, PLLC, Loveland, CO 80539, USA

**Keywords:** *Ursus* spp., Asiatic black bear, *Ursus thibetanus*, bear lameness scale, welfare assessment, lameness assessment, locomotion

## Abstract

**Simple Summary:**

Lameness scales are useful tools to identify and monitor potentially painful conditions in animals, such as acute bone, tendon, or tissue injuries as well as chronic arthritis. Pain assessment is an integral part of assessing animal welfare, especially in a zoo, aquarium, or sanctuary setting, where residents may acquire injuries or suffer from chronic conditions due to old age. While lameness scales have been developed for domestic animals, none are available for bears. Using existing scales, the Bear Lameness Scale was developed for Asiatic black bears (*Ursus thibetanus*) housed at their sanctuaries and can be used on other bear species. The scale was tested for reliability within different assessors and shown to be reliable regardless of previous experience working with bears. Having a reliable scale such as this improves the medical care given to bears in captivity, therefore improving their overall welfare.

**Abstract:**

Lameness in animals is a welfare concern as it can be an indicator of pain. A standardized bear lameness scale would significantly improve the ability of facilities that house bears to monitor, manage, and treat lameness in their animals. The Animals Asia bear rescue center in Vietnam holds over 180 rescued bears with varying health and mobility conditions as a result of the illegal bear bile trade, and a reliable lameness assessment system was needed. Bear locomotion includes a lumbering gait, which differs from domestic animal locomotion, necessitating the modification of domestic animal lameness scales, and a five-point lameness scale was developed. Professionals from various veterinary-related backgrounds scored bear lameness videos to assess interobserver reliability and the intraclass correlation coefficient indicated good to excellent reliability. A 15-min training video with examples of lameness and grades was provided before assessment. The lameness scale developed herein addresses the lack of a published lameness scale for bears, and, due to the similar locomotion of the genus, can be used on any bear species. This scale is a consistent and reliable tool for evaluating and documenting lameness in addition to monitoring response to treatment. It will benefit bear welfare by indirectly characterizing the level of pain a bear is experiencing due to lameness as well as serving to document trends in pain status.

## 1. Introduction

Lameness is a welfare concern for animals as most lame animals experience some degree of pain [1]. While welfare has been described in physical terms to measure how one is coping with the environment, an animal is considered to have good welfare if it is “healthy, comfortable, well nourished, safe, is not suffering from unpleasant states such as pain, fear and distress, and is able to express behaviors that are important for its physical and mental state” [1]. Welfare can be changed by both positive and negative experiences [2]. Therefore, lameness-associated pain can be a primary cause of compromised animal welfare and can secondarily compromise animal welfare by causing negative affective states.

Evaluating pain is challenging in animals because of its subjectivity [3] and because of species’ biology, such as hiding or masking pain [4,5]. Many zoos and aquariums are committed to conservation, education, exhibition, research [6,7], and maintain animal welfare standards, including the regular use of welfare assessments [6], which include evaluation of pain. Although welfare assessments specific for many non-domestic species are non-existent, some welfare assessments have been specifically created for wildlife housed under human care, such as for African and Asian elephants (*Loxodonta. Africana,* and *Elephas maximus*) [8,9]. Some wildlife welfare assessments are extrapolated from domestic species [10,11,12], such as in the case of farm animal welfare assessment tools adapted for gazelles (*Gazella dorcas*) and dolphins (*Tursiops truncates*) [7,9]. Bears (*Ursus* spp.) are frequently housed under human care at zoos, aquariums, and other settings worldwide and, while a species-specific welfare assessment has been developed [13], there is a need for a bear-specific lameness scale as there are none currently published.

Assessment of bear lameness requires recognition of bear locomotion and its differences to domestic animal locomotion. While bears have a lumbering gait that differs from many quadrupeds [14], the existing lameness scales for other plantigrade species can serve as a reference point for evaluating bear lameness, due in part to similarities within the gait patterns. These include the placement of the hindfoot followed by the placement of the ipsilateral forefoot, called a lateral walk, and gaits such as the walk, the running walk, and the canter. Unlike domestic animals, bear locomotion dynamics described for grizzly bears differ from other quadruped species [14] in that the bears’ weight bearing is distinct due to their high mediolateral forces [15]. Although gait has been described in the literature only in grizzly (*Ursus arctos horribilis*) and polar bears (*Ursus maritimus*)*,* it is consistent across all bear species due to bears’ plantigrade stance, long forelimbs compared to domestic animals, and similar walking patterns [16]. 

This research focused on Asiatic black bears (*Ursus thibetanus*) (ABBs) to assist in evaluating the lameness of rescued bears at the Animals Asia Vietnam Bear Rescue Center (VBRC). Musculoskeletal diseases of ABBs that are commonly associated with lameness include osteoarthritis, spondylosis of the spine, and tendon/ligament injuries [17] (personal communication, Animals Asia, 2023). Similar conditions have been found in other bear species [5,18,19,20]. There is a need to ensure that different lameness assessment observations are comparable and sufficiently consistent to ensure that the animal’s medical needs can be addressed, whether the lameness is causing acute or chronic pain [3]. There is also a need to recognize that lameness can be due to a non-painful physical condition, such as a conformational defect [21]; however, there are generally secondary musculoskeletal problems in the spine or elsewhere that result from abnormal mechanical stresses induced by the lameness that can cause chronic discomfort or pain [22]. Consequently, a standardized lameness scoring system is vital to ensure consistency when evaluating the severity of a lameness. This consistency benefits animal welfare by providing rigor when documenting lameness assessments in medical records because a consistent system supports confidence in comparisons over time and between observers. This permits evaluation of whether a lameness is becoming worse, better, or staying the same. Importantly, these temporal comparisons provide a means of evaluating analgesic efficacy and other therapeutic interventions, and also allow for consistent and clear communication between caregivers. These communications facilitate quality-of-life assessments, decision making, and evaluation of welfare states [3,5,18,19,20,21,22,23,24]. 

Domestic animal scales were evaluated to create a lameness scale for bears. The American Association of Equine Practitioners has published a horse (*Equus caballus*) lameness scale that ranges from zero to five, with zero being no perceptible lameness and five being most extreme [25]. The key features of this lameness scale include observing lameness at a walk or trot or under different circumstances, such as weight bearing, and on various surfaces. Other equine scales have incorporated audible sounds and head-bobbing, as well as lameness that is evident when the horse is trotting in circles and/or with a rider [24]. Dogs (*Canis lupus familiaris*) are another common domestic species with clinically useful lameness scoring systems, including dog lameness scales based on equine scales for evaluation [26]. However, there is no single widely accepted lameness scale for dogs. Several different dog lameness scales have been developed, with scoring systems using a one-to-six scale [27] and a different scale that scores from one to five [28] based on head and pelvic movements and stride length [28]. Most domestic animal lameness scales pair palpation with evaluations of the patient walking, trotting, and/or running [29]. 

Although lameness can be objectively measured using force-plates, pressure-sensitive mats, and treadmills, these are specialist equipment not readily available to many facilities housing bears. Another objective method for assessing lameness is to identify gait asymmetry by measuring kinematic movements, but this method also requires specialist equipment [28]. Scales based solely on visual observation have been successful for describing lameness in horses, dogs, sheep (*Ovis aries*)*,* goats (*Capra aegagrus hircus*)*,* llamas (*Lama glama*)*,* and beef and dairy cattle regardless of whether or not interrater agreement studies were conducted to support the use of these scales [8,21,25,27,30,31,32,33,34,35,36,37]. Nevertheless, subjective, or visual lameness scores must be repeatable between observers [30]. Observers trained to use a subjective lameness scale when evaluating training videos increases interobserver reliability [32]. Subjective lameness scores may not as accurately characterize lameness and cannot replace objective methods, such as force-plate analysis [32], but can be effective and are convenient and cost-effective because they require very little equipment. 

Visual observation is the only feasible method of evaluating many captive wild animal species in a non-contact setting, such as bears at the Animals Asia VBRC. Animals Asia created a bear lameness scale (Bear Lameness Scale or BLS) to evaluate lameness in the rescued bear population housed at VBRC [17]. This facility houses more than 180 bears at a time. Before arriving at the rescue center, most of the resident bears had spent over a decade on bile farms in small cages, with very poor welfare and high levels of chronic stress [38]. Due to these conditions, many rescued bears suffer from long-term diseases, such as hypertension, heart disease, dental disease, liver/gall bladder diseases, and osteoarthritis (a progressively worsening inflammation of the joint caused by the deterioration of cartilage) [15,39,40]. Most of the lameness conditions observed in rescued bears are a result of osteoarthritis, which is diagnosed by physical examination and radiographs while chemically immobilized. Acute lameness can occur from pad injuries, soft tissue injuries, and fractures [17]. Lameness is routinely treated with short- or long-term analgesics and anti-inflammatories. Therapy may need to be adjusted in response to weather fluctuations because the degree of lameness can worsen with cooler environmental temperatures, thereby necessitating increased monitoring via lameness scoring and other strategies [17]. Where indicated, additional analgesics and other therapies may be prescribed based on lameness scores and individual health conditions. 

A bear-specific lameness scale provides an option for evaluating bear lameness over multiple observations, by multiple observers, whether the lameness is acute or chronic. Repeat observations assist in assessing response to analgesics and other therapies. To specifically identify and evaluate ABB lameness, the BLS was adapted from equine and canine scales [21,25,28,41,42] due to the authors’ previous experience in working with these species. Excluded for evaluation were consideration of scales that required physical palpation of the limb, or that which required the patient to walk in a circle or back and forth as these methods are not safe or feasible with conscious bears. Also excluded were systems that looked at head or pelvic movements due to the difference in ambulation of bears, as previously discussed. Since there is little published information on bear lameness, the BLS was developed by observing lame and non-lame ABBs at the rescue center and modifying existing canine and equine lameness scales [21,25,28,41,42]. The continual and successful use of this scale at VBRC suggests acceptable interobserver reliability in identifying lameness in ABBs. Common locomotion patterns among bear species, as described above, suggest that the BLS can be generalized and applied in any setting where the evaluation of any bear species is necessary, including sanctuaries, zoos, aquariums, and in free-range settings. This study implemented a training video to prepare participants to grade bear lameness as training observers to use a lameness scale increases interobserver reliability [32]. This scale can be used to identify potentially painful lameness and monitor response to treatment. This is important because scoring mobility and lameness is an integral part of assessing welfare in bears.

## 2. Materials and Methods

### 2.1. Sample Population

The bear population chosen for this study resides at the VBRC, which was built by Animals Asia in 2006 and is located in Tam Dao National Park near Hanoi, Vietnam. The resident bears were rescued from bile farms and were rehabilitated to live the rest of their lives at the sanctuary. They have access to large semi-natural enclosures with a pool, furniture, trees, and other rotating enrichment items. Food and enrichment items are scattered throughout the enclosure to encourage bears to forage, climb, swim, and express natural behaviors. Mobility assessments were conducted upon release from night quarters into semi-natural enclosures at the beginning of the day. Upon release into enclosures, bears commonly walk or run, usually in a straight line, towards food and enrichment items. During this time, a bear’s lameness can be thoroughly evaluated; videos are routinely taken and subsequently evaluated for lameness at a later time. These videos become part of each individual bear’s permanent medical record. Although there are two bear species at the VBRC, only ABBs were chosen for evaluation. Bears with missing limbs or digits and those that were weak or ataxic were excluded from analyses based on being outside the scope for evaluation of this lameness scoring system.

### 2.2. Lameness Scale

**Figure 1:**
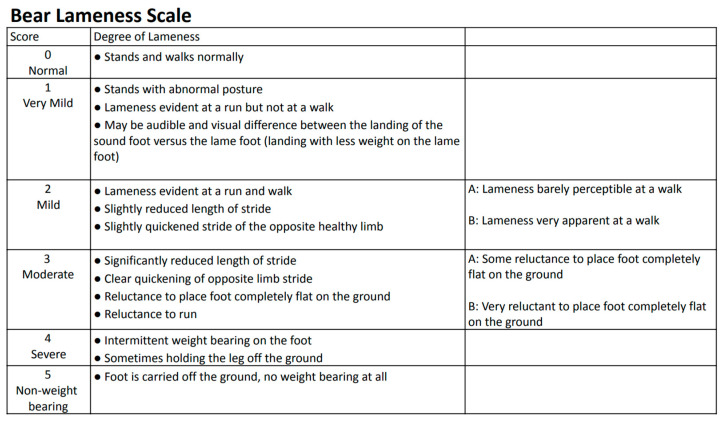
Animals Asia Bear Lameness Scale from 0 to 5.

The BLS is a numerical analog scale from zero to five, with the mild-to-moderate scores subdivided into (A) and (B) to further describe the severity of the lameness (Figure 1). The scale is as follows: 0 (normal) = stands and walks normally; 1 (very mild) = stands with abnormal posture, lameness evident at a run but not at a walk, may be audible and may have a visual difference between the landing of the sound foot versus the lame foot (landing with less weight on the lame foot); 2 (mild) = lameness evident at a run and walk, slightly reduced length of stride, slightly quickened stride of the opposite healthy limb; 2A = lameness barely perceptible at a walk; 2B = lameness very apparent at a walk; 3 (moderate) = significantly reduced length of stride, clear quickening of opposite healthy limb stride, reluctance to place foot completely flat on the ground, reluctance to run; 3A = some reluctance to place foot completely flat on the ground; 3B = very reluctant to place foot completely flat; 4 (severe) = intermittent weight bearing on the foot, sometimes holding the leg off the ground; 5 (severe) = foot is carried off the ground without weight bearing at all times during observation. Each limb is to be scored independently, and, if a score is between two numbers, the higher one should be recorded. The inclusion of further division of the mild and moderate categories is necessary due to the more general descriptions between the numerical grades, which makes scoring less accurate [31].

### 2.3. Data Collection

The VBRC bear medical records from 2017 to 2023 were retrospectively searched in order to identify the lameness videos for this study. Videos from twenty female and fifteen male bears born between 1996 and 2021 were chosen (Table 1). The list of resident bears was arranged in numerical order based on time of rescue at VBRC and the first diagnostic video in the medical file for each bear was chosen regardless of their mobility status. The process was repeated until forty videos were selected. Five bears had more than one video included in the study; their videos were taken on different dates. Of the 160 limbs to evaluate, 78% had no lameness (score of 0), 3% had very mild lameness (score of 1), 14% had mild lameness (score of 2A–B), 3% had moderate lameness (score of 3A–B), 1% had severe lameness (score of 4), and 1% were non-weight-bearing (score of 5) (Table 2). 

Videos were deemed diagnostic when (a) the bear in the video had taken at least four consecutive steps (walking or running) in a straight line on short grass or concrete; (b) the bear did not stop to sniff or eat; and (c) all four legs are visible. Videos contained a mixture of side (taken from the ground) and roof views (taken from the roof of the bear house, overhead). Eighteen videos were taken from the side and twenty-two were taken from the roof (Table 1). Videos had no sound and had a duration of nine seconds to one minute and nine seconds. Arrows were inserted into the videos for trainees to identify the bear to be assessed if multiple bears were in the video. Videos were uploaded onto an unlisted YouTube channel and then inserted into a Google Forms document where assessors could access them. According to their published guidelines, an Institutional Animal Care and Use Committee (IACUC) was not required because this study was based on retrospective clinical data at the VBRC [43]; the UC Davis Institutional Review Board (IRB) determined that an IRB review is not required for this study [44].

### 2.4. Training and Observers

A 15-min recorded presentation with descriptions and sample videos explaining the BLS was provided to train participants. The ability to replay the training video and the lameness videos was unlimited; observers could return and edit previous scores and there was no set time limit. Twenty observers with a range of veterinary-related careers, contacted through veterinary and animal care social media groups, voluntarily evaluated the selected videos of bears to assess interobserver agreement using the BLS. Participants (*n* = 20) included veterinarians (*n* = 17), veterinary technicians (*n* = 2), and a researcher (*n* = 1). Of the veterinarians, seven were small-animal-focused, five were mixed-focused, three were large-animal-focused, and two were zoo-focused. Of the veterinary technicians, one was small-animal-focused and the other was mixed-focused. The researcher was zoo-focused. All raters were scored together as this scoring system was designed to be used by any animal care professional.

Following the completion of all assessors’ scores and consideration of their feedback regarding video quality, the videos were graded and divided into three categories: (1) Diagnostic-Quality; (2) Mediocre-Quality; and (3) Poor-Quality. Diagnostic-Quality videos (*n* = 20) allowed viewers to see the bear from the side and the back and the feet were clearly visible. Mediocre-Quality videos (*n* = 15) were not as clear, without all four feet being visible, but with the lame leg visible and identifiable. Poor-Quality videos (*n* = 5) were generally too far away, with feet not clearly visible, or the video not offering a view of the bear from the side.

### 2.5. Statistical Analysis

Statistical analyses were performed using RStudio version 4.1.1 [45]. Intraclass correlation coefficient (ICC) was used to determine the interrater reliability of bear lameness scoring conducted by multiple independent raters. A two-way random effects model was used with absolute agreement, an average measure, a 95% confidence level, and an alpha of 0.05. The outcome variable was the rater’s score, the fixed effect was the individual bear leg being scored, and both the subjects and raters were included as random effects. ICC was first calculated for all 40 videos, then excluding the Poor-Quality videos (i.e., Diagnostic-Quality and Mediocre-Quality videos only), and finally calculated for only the Diagnostic-Quality videos. Scores less than 0.5 indicate poor reliability, scores between 0.5 and 0.75 indicate moderate reliability, scores between 0.75 and 0.9 indicate good reliability, and scores greater than 0.9 indicate excellent reliability [46].

## 3. Results

Observers (*n* = 20) with differing experiences in evaluating lameness successfully completed training and review of lameness videos (*n* = 40). The ICC for all 40 videos (160 legs) yielded an ICC of 0.848 (95% confidence interval 0.81, 0.881, *p* < 0.05). The ICC for the Diagnostic- and Mediocre-Quality videos (*n* = 35, 140 legs) was 0.836 (95% confidence interval, 0.792, 0.874, *p* < 0.05). The ICC for the Diagnostic-Quality videos (*n* = 20, 80 legs) was 0.869 (95% confidence interval 0.822, 0.907, *p* < 0.05) [46,47]. 

An interobserver rating score between 0.75 and 0.9 indicates good reliability, and scores greater than 0.9 indicate excellent reliability [46]. Thus, using video recordings where the bear can be seen from the front and side and the limbs can be visualized, the BLS can reliably be used between observers of different backgrounds to identify and monitor lameness. 

## 4. Discussion

Evaluating the interrater reliability between observers showed that the BLS can be used reliably to identify and monitor lameness in ABBs. The BLS was designed to be used as a tool that allows consistent and sequential assessments of bear lameness among evaluators regardless of previous experience evaluating bears. The twenty assessors demonstrated that our training in the use of the BLS was effective and that it is readily applicable to bears for professionals that were not in the zoo/wildlife sector, including those with minimal previous lameness assessment experience for any species. As the majority of the observers were in the veterinary field, more research is needed to rate reliability within non-veterinary animal care personnel. 

The BLS has the advantage of not requiring specialized tools other than video recording and review capability. Videos can be acquired during basic husbandry without requiring the bear to undertake directed locomotion. A key consideration in using previously recorded videos for lameness assessment is that the video must be of high quality, the bear must be close enough for evaluation, the view can be taken from the ground (side view) or from the roof (overhead view), and all four legs and feet must be visualized in the video. These are likely not to be problematic issues for live scoring [35], so, if these criteria are met, this scoring system should produce reliable results. This excellent reliability for mobility scoring with diagnostic-quality videos indicates that zoos, aquariums, and sanctuaries can accurately identify and characterize lameness in bears. 

Although there are conflicting results in the literature about the effectiveness of training on interrater reliability [30,32,35], our results suggest that our training video was sufficiently intensive to achieve good interobserver reliability. This is important because lameness assessments can be the basis for the monitoring and treatment of acute and chronically painful musculoskeletal conditions [48,49]. Bears are known to be “stoic”, meaning that they often do not show external signs of pain [5], making lameness assessments more important for regular monitoring. These lameness scores and their trends over time are the foundation for managing and treating lameness [24] and become a part of the animals’ permanent medical record; they are useful for assessing response to medications and other therapies. 

The BLS can be an important tool as a part of quality-of-life assessments, especially for older bears with chronic conditions. Animals in captivity suffer from chronic conditions such as osteoarthritis as they live much longer than their wild counterparts [50,51]. The bear residents at the VBRC can live more than 30 years [17] and, during the bears’ extended residence at the facility, veterinary and animal care staff turnover occurs. The reliability and consistency of the BLS enables uniform lameness assessments over time as new staff review and add to each bear’s medical records. This is essential for maintaining good patient care and welfare as consistency over time provides the ability to evaluate treatments with a reliable assessment tool [52] as well as to assess quality of life [53]. This makes the BLS a valuable and practical method of assessing lameness for zoos, sanctuaries, and other facilities that house captive bears.

Limitations of the BLS include its requirement for training and the element of subjectivity. Additionally, the BLS cannot be used to assess bears with weakness or ataxia, nor for bears missing digits or limbs due to their changed mobility. Validation of this scale with objective measures would be ideal but requires additional funding for equipment. Nevertheless, this tool has proven to be clinically useful at the VBRC. While Shine, et al. were able to train four grizzly bears to walk across a force-plate at Washington State University under controlled laboratory conditions as part of research on bear locomotion [14], this approach would not be practical in a rescue sanctuary of more than 180 bears due to time and logistic constraints. Facilities with adequate funding for equipment, sufficient staffing, and few bears could potentially use force-plate analysis or similar objective strategies for assessing bear lameness. Most facilities will not have such resources and will need to rely on the BLS as the most reliable [30] and practical way to monitor bear lameness. 

Although this scale was developed for ABBs, it has been used on Malayan sun bears (*Helarctos malayanus*), Tibetan brown bears (*Ursus arctos pruinosus*), and Eurasian brown bears (*Ursus arctos arctos*) at Animals Asia’s sanctuaries. The similar anatomy and locomotion patterns of these bears [16] allowed the seamless use of the scale to monitor acute or chronic lameness. Although there has been no interobserver reliability performed, the BLS would likely be of value when assessing the lameness of other bear species in other facilities that house bears in captivity, which inherently elevates the level of care and welfare the bears experience. 

The methods used to develop the BLS may be valuable as a model for evaluating lameness in other carnivore species in zoos, sanctuaries, and other settings where visual lameness assessments are the sole diagnostic method available. The numerical analog scale and criteria for diagnosis provide a semi-quantitative methodology for describing lameness severity and allow comparisons between observers and over time. The personnel training method that we used, use of video from differing perspectives, and assessment of video quality increase the consistency and reliability of observations by different personnel and over time. Independent lameness assessment of each limb and corresponding documentation also provide a standardized method for diagnosis and are useful for identifying temporal trends. Therefore, while the BLS may not be applicable to all carnivore species, our methodological approach may be valuable for lameness assessment for species without established lameness scales.

## 5. Conclusions

The BLS was created for ABBs by modifying domestic animal lameness scales. The BLS has a high interrater reliability among assessors with a variety of background animal care experience and training. A reliable visual lameness scale is important for identifying and monitoring lameness and supports treatment and monitoring for alleviation of pain in bears. Therefore, the BLS is an important tool for supporting the welfare of bears under human care.

## Figures and Tables

**Table 1:** Vietnam Bear Rescue Center bears with videos of lameness that were evaluated.

Bear	Sex	Est DOB	Date of Rescue	Lameness Scores of 4 Limbs ^1^ Right Front, Left Front, Right Hind, Left Hind	Video Perspective Roof ^2^/Side ^3^	Video Quality Ranking ^4^
V027 Bubu	M	2006	31-Jul-2009	0, 2A, 0, 0	Roof	3
V031 Zebedee	M	1996	24-Nov-2009	2B, 0, 0, 0	Roof	2
V044 Leila	F	2001	21-Jan-2010	2B, 0, 0, 0	Side	1
V048 Nicole	F	2003	21-Jan-2010	0, 2A, 0, 2A	Roof	1
V208 Sumaya	M	2017	28-Jun-2019	0, 0, 0, 0	Roof	1
V053 Hope	F	2006	27-Apr-2010	0, 0, 0, 2A	Roof	3
V234 Star	M	2021	21-Dec-2021	0, 0, 0, 0	Side	2
V125 Salsa	F	2005	6-May-2015	0, 2A, 0, 0	Side	1
V127 Oscar	M	2000	28-Jun-2015	2B, 0, 0, 0	Side	1
V151 Quang Yen/Royale	M	2002	22-Sep-2015	0, 3A, 0, 0	Roof	2
V153 Kesha	F	2003	21-Oct-2015	0, 0, 0, 0	Roof	1
V154 Gloria	F	2000	21-Oct-2015	0, 0, 2A, 0	Roof	2
V158 Arthur	M	2002	22-Oct-2015	0, 0, 0, 3A	Roof	1
V239 Tuyet	F	2005	21-Dec-2021	0, 0, 0, 2B	Side	2
V008 Mischa	M	2006	24-Apr-2008	0, 2A, 0, 0	Side	2
V014 Irwin Junior	M	2008	24-Jun-2008	0, 0, 0, 0	Roof	2
V015 Leika	F	2004	9-Aug-2008	0, 0, 0, 0	Roof	3
V249 King Darbs	M	2002	7-Jul-2022	0, 0, 0, 1	Side	2
V003 Mausi	F	2007	31-May-2007	0, 0, 2A, 0	Roof	2
V040 Scrumpy	M	2004	21-Jan-2010	0, 0, 1, 1	Roof	2
V008 Mischa	M	2006	24-Apr-2008	0, 2B, 0, 0	Side	1
V137 Irene	F	2002	26-Jun-2015	0, 0, 0, 0	Side	1
V079 Faith	F	2007	26-Apr-2011	0, 0, 0, 2B	Roof	1
V136 Max	M	2001	26-Jun-2015	3B, 3B, 0, 0	Roof	1
V089 Yulgibar	M	2007	15-Sep-2011	0, 0, 2A, 2A	Side	1
V096 Milagro	M	2004	3-Dec-2011	0, 2A, 0, 2A	Side	1
V097 Mary	F	2004	3-Dec-2011	0, 0, 4, 0	Side	1
V104 Luna	F	2004	3-Dec-2011	0, 0, 2B, 2B	Side	1
V113 Nora Jamjack	F	2013	12-Apr-2013	0, 0, 0, 0	Roof	1
V014 Irwin Junior	M	2008	25-Jun-2008	0, 0, 0, 0	Roof	2
V160 Bong Bong	F	2002	22-Oct-2015	0, 5, 0, 0	Side	2
V190 Anh Sang	F	2002	11-Dec-2017	0, 0, 1, 1	Side	1
V195 Kim	F	2002	21-Aug-2018	0, 0, 0, 4	Side	1
V197 Mai	F	2002	31-Aug-2018	0, 2A, 0, 0	Side	1
V027 Bubu	M	2006	31-Jul-2009	0, 0, 0, 0	Side	2
V030 Easy	F	2000	13-Oct-2009	0, 2A, 0, 0	Side	3
V031 Zebedee	M	1996	14-Nov-2009	0, 0, 0, 0	Side	1
V040 Scrumpy	M	2004	21-Jan-2010	0, 0, 0, 0	Roof	3
V058 Shima	F	2010	1-Jul-2010	0, 0, 0, 2A	Side	2
V059 Fritz	M	2007	1-Jul-2010	0, 0, 0, 0	Side	2

^1^ Lameness score assigned by trainer; ^2^ Roof = overhead view from the roof, ^3^ Side = side view, taken from the ground. ^4^ 1= Diagnostic Quality; 2 = Mediocre Quality; 3 = Poor Quality.

**Table 2:** Number of limbs in each lameness group with their associated scores.

	Lameness Scores							
	0 Normal	1 Very Mild	2a Mild	2b Mild	3a Moderate	3b Moderate	4 Severe	5 Non-Weight-Bearing
Number of limbs	125	5	15	8	2	2	2	1

## Data Availability

Data available on request due to privacy restrictions. The data presented in this study are available on request from the corresponding author. The data are not publicly available to protect the names and contact information of the assessors.
